# Automatic Assignment of Molecular Ion Species to Elemental Formulas in Gas Chromatography/Methane Chemical Ionization Accurate Mass Spectrometry

**DOI:** 10.3390/metabo13080962

**Published:** 2023-08-19

**Authors:** Shunyang Wang, Luis Valdiviez, Honglian Ye, Oliver Fiehn

**Affiliations:** 1 Department of Chemistry, University of California, Davis, CA 95616, USA; 2 West Coast Metabolomics Center, University of California, Davis, CA 95616, USAhlye@ucdavis.edu (H.Y.)

**Keywords:** chemical ionization, quadrupole time-of-flight, compound identification

## Abstract

Gas chromatography–mass spectrometry (GC-MS) usually employs hard electron ionization, leading to extensive fragmentations that are suitable to identify compounds based on library matches. However, such spectra are less useful to structurally characterize unknown compounds that are absent from libraries, due to the lack of readily recognizable molecular ion species. We tested methane chemical ionization on 369 trimethylsilylated (TMS) derivatized metabolites using a quadrupole time-of-flight detector (QTOF). We developed an algorithm to automatically detect molecular ion species and tested SIRIUS software on how accurate the determination of molecular formulas was. The automatic workflow correctly recognized 289 (84%) of all 345 detected derivatized standards. Specifically, strong [M − CH_3_]^+^ fragments were observed in 290 of 345 derivatized chemicals, which enabled the automatic recognition of molecular adduct patterns. Using Sirius software, correct elemental formulas were retrieved in 87% of cases within the top three hits. When investigating the cases for which the automatic pattern analysis failed, we found that several metabolites showed a previously unknown [M + TMS]^+^ adduct formed by rearrangement. Methane chemical ionization with GC-QTOF mass spectrometry is a suitable avenue to identify molecular formulas for abundant unknown peaks.

## 1. Introduction

The identification and characterization of metabolites are the heart of metabolomics studies. Gas chromatography–mass spectrometry (GC-MS) is a mature technology for small metabolites profiling, enabling the separation and detection of compounds with a wide coverage of chemical classes [1] and high reproducibility [2]. A standard ionization method that has been widely adopted in order to compare results across different instruments and labs is 70 eV electron ionization (EI) [3]. For analyzing small molecules and volatile compounds to provide fragmentation patterns, 70 eV EI is particularly effective. Meanwhile, low energy electron ionization leads to less sensitivity and fewer fragmentations [4,5]. To help identify unknown mass spectra against reference spectra, large mass spectral libraries are available for compound identification, such as the NIST EI library [6], the MassBank of North America [7], and the Human Metabolome Database [8]. When combined with automated data processing, spectra matching has helped the rapid and comprehensive analysis of metabolomics samples. However, the number of reference spectra is limited by the availability of the standards. While over 116 million known compounds have been recorded in PubChem (August, 2023), only 347,000 unique compounds have EI mass spectra in the NIST library [6]. The in-silico generation of reference spectra, including quantum chemistry molecular dynamics simulation [9,10], still has difficulties in prediction accuracy. Compound annotation based on calculating fragmentation trees and fingerprint prediction for mass spectra [11] is an alternative strategy that does not require a reference spectral library. However, a critical aspect of calculating fragmentation trees is the determination of elemental formulas for the molecular ion species observed in mass spectra. Yet, because EI is a hard ionization technique leading to strong ionization and fragmentation, molecular ion adducts are usually of low abundance or absent, especially when using classic trimethylsilylation (TMS). TMS derivatization is a classic reaction in GC-MS screening to improve the volatility and stability of analytes in the gas phase. For TMS derivatives in EI, a methyl group loss from the TMS group ([M − CH_3_]^+^) is regularly observed in addition to a neutral loss of TMSOH ([M − TMSOH]^+^) [12], while the molecular ion itself is often of low abundance or not observed. Without accurately knowing the molecular masses of unknown metabolites, calculating fragmentation trees for structural identifications is impossible.

Alternatively, chemical ionization [2] (CI) is a softer technique than electron impact. Unlike the energetic electron impact in EI, the CI process involves an interaction of analyte molecules and reagent ions. It usually obtains molecular adducts at higher relative abundance and has been successfully used for compound identifications [13]. In chemical ionization, the reagent gas molecules (usually methane, ammonia or isobutane) are first ionized and then react to ionize neutral analyte molecules. Compared to 70 eV EI, CI transfers less energy due to the exothermicity of ion–molecule reactions [3], leading to a higher probability of retaining molecular ion adducts and fewer fragmentations than EI. For methane CI, the following complex reactions have been described to produce typical molecular ion adducts [14,15]:

$$M+{CH}_{4}^{+}\to {CH}_{4}+M^{+}$$

$$M+{CH}_{5}^{+}\to {CH}_{4}+{\left[M+H\right]}^{+}$$

$$M+{CH}_{3}^{+}\to {CH}_{4}+{\left[M-H\right]}^{+}$$

$$M+{C_{2}H}_{5}^{+}\to {\left[M+{C_{2}H}_{5}^{\;}\right]}^{+}$$

$$M+{CH}_{2}^{+}+{2CH}_{4}^{\;}\to {\left[M+{C_{3}H}_{5}^{\;}\right]}^{+}+{2H}_{2}+H^{\cdot }$$

We tested the hypothesis that this formation of a series of predictable adducts could assist in automatically assigning the molecular ions in GC-chemical ionization MS. Additionally, CI’s soft ionization nature could facilitate the analysis of labile and polar compounds that might be prone to extensive fragmentation or ionization inefficiency under EI conditions. Combined with the determination of accurate masses using quadrupole time-of-flight mass spectrometry and advanced software, one should be able to correctly assign molecular formulas to unknown compounds. In this paper, we explored the feasibility of using automatic pattern analysis for recognizing molecular ion species in GC-CI-QTOF MS and then used that information to obtain elemental formulas. We performed these analyses on a large range of metabolites under trimethylsilylation conditions, as used in untargeted GC-MS metabolomics studies.

## 2. Materials and Method

### 2.1. Data Acquisition

To build a GC-CI-QTOF mass spectral test library, 1 mg of each metabolite standard was dissolved in a 1 mL solution of methanol/water/isopropanol in a ratio of 5:2:2. To minimize data acquisition time, 20 μL of each standard was combined into mixtures of 20 non-isomeric compounds. Subsequently, the mixtures were evaporated to dryness and derivatized by methoximation and trimethyl silylation as published previously [1]. O-methyl hydroxylamine hydrochloride solution from Sigma-Aldrich, in conjunction with pyridine, was employed for methoximation, while N-methyl-N-trimethyl silyl trifluoroacetamide (MSTFA) from Sigma-Aldrich facilitated trimethyl silylation. Retention index markers of C8–C30 linear chain fatty acid methyl esters (FAME markers) were added to the MSTFA. Then, 100 μL samples were transferred to autosampler vials and 1 μL of the resulting solution was injected at a 25 s spitless time (more details in Table 1).

### 2.2. Data Analysis and Molecular Assignment Algorithms

SIRIUS and CSI:FingerID [11] were used to predict molecular formulas. In the SIRIUS parameter tab, we selected the Q-TOF instrument and MS2 mass accuracy 10 ppm options. We saved the top 10 candidates for each test and only exported formula prediction results. Because SIRIUS and CSI:FingerID were developed for MS2 spectra, we deduced the molecular ion information from chemical ionization MS1 patterns to provide this precursor ions information as input in MS file format (a special input file format for SIRIUS and CSI:FingerID). A Python code was developed based on characteristic ion patterns of chemical ionization mass spectra to automatically assign molecular ions to the mass spectra. Python tools were used to convert MSP text format of mass spectra into comma-separated values format, MGF format and MS format. A further Python tool calculated derivatized molecular formula and molecular weight from the PubChem active hydrogen count, and a third tool evaluated the accuracy of predictions. All code is available at https://github.com/Shunyang2018/EICI (accessed on 11 August 2023 ). 

## 3. Results

### 3.1. CI Pattern of Molecular Ion Species

**Figure 1:**
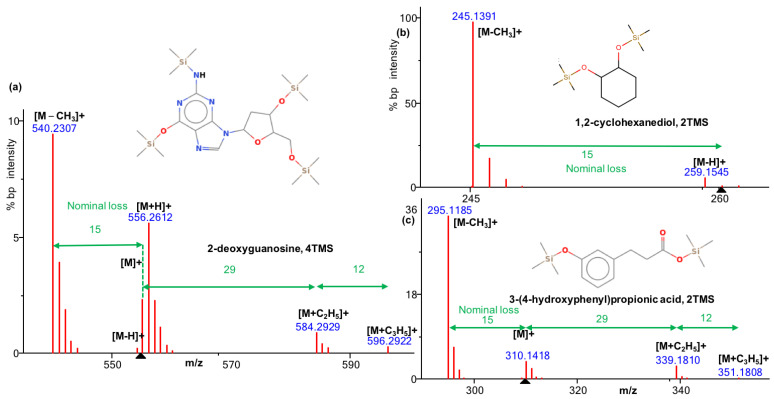
Examples of molecular ion species patterns in methane chemical ionization GC-QTOF MS. (**a**) CI pattern of 2′-deoxyguanosine, 4TMS, [M + H]^+^ with [M + C_2_H_5_]^+^, and [M + C_3_H_5_]^+^; (**b**) CI pattern of 1,2-cyclohexanediol, 2TMS, and [M − H]^+^; no further adducts detected; (**c**) CI pattern of 3-(4-hydroxyphenyl) propionic acid, 2TMS, [M]^+^ with [M + C_2_H_5_]^+^, and [M + C_3_H_5_]^+^; and black triangle on x axis: MS1 precursor ions.

We first manually investigated CI mass spectra and confirmed the frequent observation of a pattern of ions derived from the molecular ion: [M − CH_3_]^+^, [M − H]^+^, [M]^+^, [M + H]^+^, [M + C_2_H_5_]^+^, and [M + C_3_H_5_]^+^. [M − CH_3_]^+^ is not a characteristic peak observed exclusively in CI spectra. This ion arises due to the neutral loss of a methyl group from the derivatized analytes, particularly those involving trimethylsilyl derivatization. Thus, [M − CH_3_]^+^ also exists in EI spectra. Notably, in both EI and CI spectra, the [M − CH_3_]^+^ is often observed as a base peak ion (bp, the most abundant peak in the spectrum), especially for aromatic or nitrogenous compounds. This phenomenon occurs because of the stability of the resulting fragment, while molecular ion species [M − H]^+^, [M]^+^, and [M + H]^+^ were presented at variable abundance but usually at larger than 5% bp intensity. Exceptions were found for [M + C_2_H_5_]^+^ and [M + C_3_H_5_]^+^, which were mostly found at <5% bp intensity. Occasionally, additional ions were observed at lower intensity, as described before [10,11]. A Python script based on those fragmentation patterns was developed to identify CI patterns by finding these isotopic ion groups and utilizing the nominal mass difference between them (Figure 1). The molecular mass detection of [M − H]^+^, [M]^+^, and [M + H]^+^ resulting from the pattern recognition was used as precursor mass information and combined with the CI spectrum as the MGF format to be used for the SIRIUS + CSI:FingerID [11] software. SIRIUS and CSI:FingerID are usually employed for tandem MS/MS spectra annotation but were used here to predict the molecular formula, including silicon as a mandatory element for TMS-derivatized metabolites.

### 3.2. Overall Detection Rate of Molecular Ion Species in GC-CI-QTOF MS

We probed 369 standards (Supplementary Data S1) and acquired them at high concentrations in GC-methane CI-QTOF MS. Employing a strategy of amalgamating these standards into mixtures of 20 non-isomeric compounds, we successfully detected 323 unique standards via manual curation. Furthermore, 345 TMS-derivatized versions of these compounds were detected, including 22 spectra originating from derivatives with a different number of TMS groups. It is noteworthy that 46 compounds remained undetected even after manual curation (Supplementary Data S1). The non-detection of these 46 compounds can be attributed to low CI efficiency or chemical properties not suited for the gas chromatography process. To test the automatic molecular ion assignment algorithm, CI spectra were then processed by the CI pattern algorithm. We compared this result with manual curation to find less abundant compounds that might not have fit the algorithm pattern. We detected molecules with molecular mass up to 991.440 Da (isomaltose, eight TMS). Water loss fragment ions were observed in 6% of the detected compounds, with up to 15% of the detected molecules for the class of amino acids and peptides (Supplementary Data S1). Table 2 gives an overview on the diversity of chemical classes included in the mixtures using the ClassyFire software [17]. Purine and pyridines, fatty acids, indoles, carboxylic acids, and hydroxy acids were well covered in CI detection, while only half of the tested organonitrogen compounds were positively identified in our tests (Table 2). Within the carboxylic acids and derivatives class, we detected 73 out of 80 injected molecules in the subclass of amino acids, peptides, and analogues. Four dipeptides and tripeptides (ophthalmic acid, Asp-Glu, Gly-Tyr, and Gly-Pro) were detected in the CI mode with up to four TMS derivatization groups (Supplementary Data S1). Carbohydrates, classified by the ClassyFire software as organooxygen compounds, were often true negatives even in manual investigations (Table 2), most likely because these compounds bear many TMS derivative groups. For these compounds, even soft chemical ionization might lead to the fragmentation of molecular ion adduct species and therefore a loss of molecular ion information. Prenol lipids and steroids were also rarely detected in CI mode (Table 2), likely because of a lack of ionization efficiency in CI mode compared to classic electron ionization. For most TMS-derivatization products, retention index information was neither available in MassBank.us nor NIST20 libraries. We therefore used wide retention index windows to find the TMS-derivatized standards within the mixtures. Validation measurements showed that accurate mass-based peak findings led to a 1.3% occurrence of false positive annotations (five compounds). When comparing the results of automatic assignments with manual curation, we found that 37 molecules presented additional ions at higher *m*/*z* values than [M + C_3_H_5_]^+^. This discrepancy to the molecular adduct pattern prevented the automatic deduction of the molecular ion (Supplementary Data S1). For an additional 14 mass spectra, CI ion intensity patterns were too low to be distinguished from noise ions, again causing false negative recognition of the molecular ion by the automatic algorithm. We also confirmed a previous report showing that the sensitivity of GC-MS with chemical ionization is about 20-fold lower than GC-MS electron ionization mass spectrometry [18]. This shortcoming imposes constraints on the utilization of chemical ionization for the identification of unknown metabolites, confining its practicality to compounds of higher abundance.

**Figure 2:**
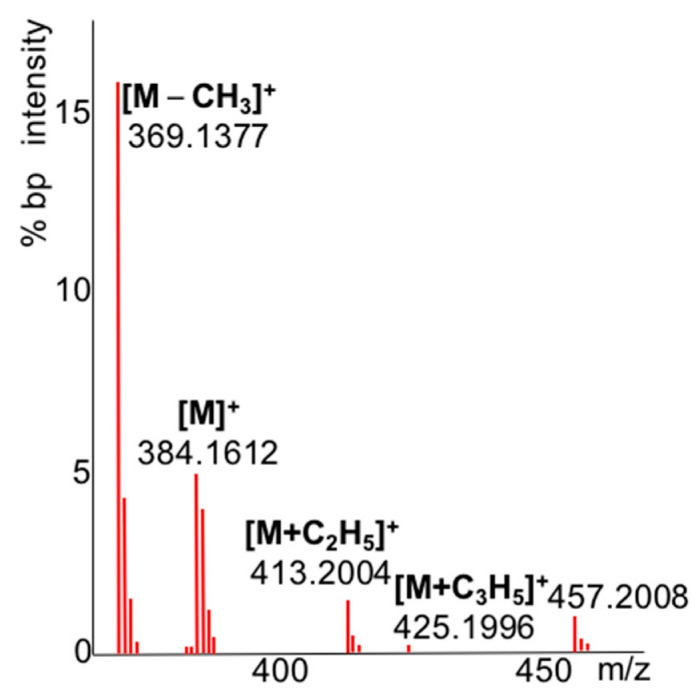
Methane CI QTOF MS spectrum of the molecular ion species region of 3,4-dihydroxyphenylacetic acid 3 TMS.

Overall, we detected 345 unique standards after manual curation, with an average mass of 345 ± 160 Da and an average mass error for the [M − CH_3_]^+^ ion species of 0.001 ± 0.0008 Da (Supplementary Data S1). These data showed excellent mass accuracy for this instrument, with only 2.8 ppm error, which led us to expect high success rates for calculating elemental formulas. Of the molecular ion species clusters ([M − H]^+^, [M]^+^, and [M + H]^+^) that were automatically detected by the algorithm, 70% had the highest intensity for [M + H]^+^ while many derivatives were surprisingly detected with the highest abundance as [M − H]^+^ species (7%) or as [M]^+^ species (4%) (Table 3). Interestingly, 14% of the [M − CH_3_]^+^ ion species were not recognized by the algorithm but were only found by manual investigations. Figure 2 shows the spectrum for 3,4-dihydroxyphenylacetic acid as an example spectrum that was rationalized manually, but that was not automatically annotated by the algorithm due to the presence of unexplained ion species above the maximum [M + C_3_H_5_]^+^, here at *m*/*z* 457. In the remaining 289 cases for which we automatically found [M − CH_3_]^+^ ion species, we also detected corresponding [M + C_2_H_5_]^+^ ion species 90% of the time, while [M + C_3_H_5_]^+^ ion species were detected 84% of the time. Overall, the combined pattern analysis of all signature ion species led to high confidence for an automatic detection of molecular ions in GC-QTOF MS.

Within the 51 CI spectra that did not yield automatic annotations of [M − 15]^+^ ion species, we found many examples that followed the same pattern as given in Figure 2. We rationalized these new ion species as previously unreported [M + TMS]^+^ ions and give mass errors for three examples in Table 4. These examples unequivocally support the interpretation of these ion species, with excellent mass accuracies. Because the molecules themselves do not bear additional exchangeable, acidic protons, we concluded that these species were likely generated by intermolecular ion rearrangements of [M] ^• +^ ions with TMS^•^ radicals that were cleaved from molecules within the CI reaction zone, supported by the high concentration of analyte ions used in our test cases.

### 3.3. Automatic Calculation of Elemental Formulas

**Figure 3:**
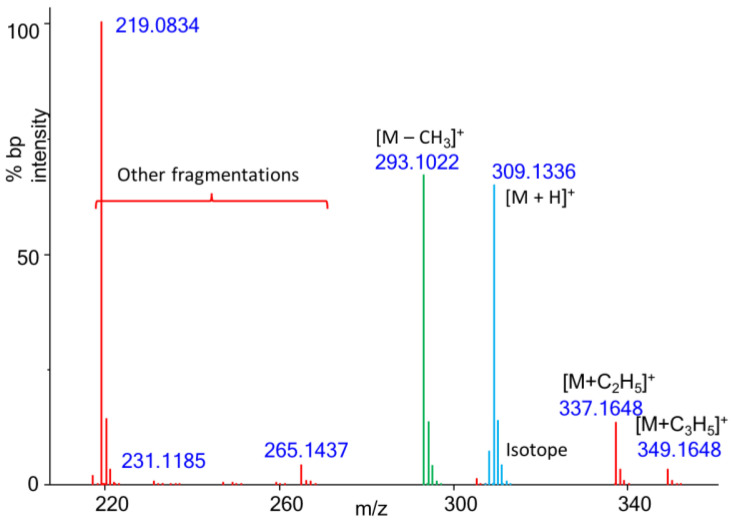
Automatic calculation of molecular formulas by Sirius/CSI:Finger ID software using CI-QTOF MS data. Example CI spectra of 2-hydroxycinnamic acid, 2TMS. Green: [M − CH_3_]^+^ isotope cluster; blue: molecular ion species summarizing [M − H]^+^, [M]^+^, and [M + H]^+^; red: other fragments in CI-QTOF MS spectrum.

Obtaining the correct molecular formula is the starting point for identifying unknown compounds in metabolomics. SIRIUS + CSI:FingerID was designed to interpret tandem mass spectrometry (MS/MS) consisting of both MS1 precursor ions and MS/MS fragment ions. SIRIUS employs fragmentation trees derived from mass spectral neutral loss data, augmenting their isotope pattern analysis to enhance the accuracy of computed molecular formulas. To extend the software’s application to GC-CI-QTOF MS spectra, we adapted the file formats to incorporate molecular mass data produced by our automated pattern-recognition algorithm. We then tested which ions were best suited to calculate correct elemental formulas in SIRIUS software by probing the most abundant [M − CH3]^+^ characteristic ion, the molecular ion species recognized by our pattern algorithm ([M+]^+^, [M − H]^+^ or [M + H]^+^), or by using the isotope information in an overall combination with either molecular ion species and the [M − CH3]^+^ characteristic ion (Figure 3). We achieved this differentiation by either separating MS1 information as input (blue labeled ions in Figure 3) or excluding that information and only relying on the overall CI-QTOF fragment masses (green and red labeled ions in Figure 3). Surprisingly, adding isotope distribution analysis to the accurate masses for elemental formula calculations dramatically worsened the accuracy (Figure 3, Table 5) compared to calculations that did not use isotope ratio information. This result is due to complex reactions in chemical ionization that led to mixtures of molecular ion species and their natural isotope abundances (see Figure 3). Here, the ^13^C natural isotope of the [M − H]^+^ ion would be measured together with the ^12^C monoisotope ion of the [M]^+^ ion because their accurate masses would be too close to be resolved with the QTOF MS instrument used here.

Conversely, the ^13^C-natural isotope of the [M]^+^ ion species also contributes to the accurate mass and isotope abundance measurements for the [M + H]^+^ ion (see Figure 1). Likely for this reason, using the accurate mass of the molecular ion species with all fragment ions yielded only 60.7% correct top-hits (Table 5, Supplementary Data S1). In comparison, using all fragment ions, specifically when identifying the [M − CH_3_]^+^ species, gave 71.4% correct top-hits and 87% correct hits within the top three ranked formulas. Here, the higher abundance of [M − CH_3_]^+^ certainly improved measurement accuracy. However, this species is also void of isotope contributions from other ion species because it can only derive from a methyl neutral loss of the corresponding ^12^C monoisotopic [M]^+^ ion.

When only querying [M − CH_3_]^+^ species, Sirius software did yield a hit for 6.9% of the test cases. Nevertheless, in six of those cases, both [M]^+^ and [M + H]^+^ ion species returned the correct formula as top-hit. For most other cases, Sirius software did not return any hits when the formula calculation of [M − CH_3_]^+^ ions failed. Hence, in summary, more than 70% of the automatically detected molecular ion species resulted in the correct formula as top-hit when considering [M − CH_3_]^+^ species and adding [M]^+^ and [M + H]^+^ ion calculations for confirmation in cases where [M − CH_3_]^+^ calculations failed. If researchers widen their search to the top three formula hits in compound identification workflows, more than 87% of these formulas would be expected to be correct (Figure 3, Table 5). From the correct elemental formula, there are established algorithms to obtain 2D structure identification, as previously published [9].

## 4. Conclusions

We showed that GC-chemical ionization-QTOF MS can be used to automatically identify molecular ions and correctly assign elemental formulas. However, the lower sensitivity of chemical ionization means that routine metabolomics screening should still utilize electron ionization GC-MS. To test the mass accuracy of a commercial GC-QTOF under methane chemical ionization and its suitability to automatically detect molecular ions and elemental formulas, 369 metabolite standards were used. Ninety-five percent of the detected trimethylsilylated analytical standards provided high-quality CI spectra. We devised a novel algorithm that automatically searched patterns for molecular ion species, adducts, and neutral loss of methyl groups and found that this algorithm correctly found 84% of all detected test molecules. Using those accurate masses in SIRIUS + CSI:FingerID software yielded 91% correct formulas in the top five hits, and more than 71% correct formulas were retrieved as top-hit. Overall, we recommend using methane chemical ionization with GC-QTOF MS mass spectrometry as a viable route towards the identification of abundant GC-MS peaks.

## Figures and Tables

**Table 1:** Details of data acquisition parameters for the Fiehn Lib GC/MS libraries.

Gas Chromatograph	Agilent 7890A GC System
Mass Spectrometer	7200 accurate mass QTOF mass spectrometer
GC column	DB5 MS column 30 m + 10 m integrated guard, 0.25 mm id, 0.25 μm film
GC parameters, injection	1 μL in 25 s splitless mode at 250 °C
GC parameters, separation	Initial temperature of 60 °C with a hold time of 0.5 min, a temperature ramp of 10 °C/min to 325 °C, and a final hold time of 10 min at 325 °C.
EI ion source	temperature, 230 °C; energy, 70 eV
Chemical ionization	Ion source 300 °C; CI electron energy 135 eV; CI methane gas flow rate 20%
MS parameters, tuning	Autotune using FC43 (Perfluoro tributylamine)
MS parameters, data acquisition	*m*/*z* 50–1200 at 5 Hz scan rate and 750 V detector voltage in both electron ionization (EI) mode and chemical ionization (CI) mode
MS data processing	Peak detection, deconvolution by MS-DIAL 4 [13,16]

**Table 2:** Chemical classes detected by CI mode using ClassyFire software for classes with *n* > 5 molecules.

Class	#	Detected by CI (%)
Carboxylic acids and deriv.	77	83.1
Organooxygen compounds	47	55.3
Benzene and substituted deriv.	35	77.1
Fatty acyls	29	89.7
Phenols	26	73.1
Indoles and deriv.	15	86.7
Organonitrogen compounds	11	63.6
Hydroxy acids and deriv.	9	88.9
Phenylpropanoic acids	8	87.5
Prenol lipids	8	25.0
Cinnamic acids and deriv.	7	71.4
Pyridines and derivatives	7	100.0
Steroids and steroid deriv.	7	14.3
Purine nucleosides	6	100.0

**Table 3:** Count and molecular ion species of derivatized standards that were recognized automatically by the pattern algorithm.

[M]^+^	15	4%
[M − H]^+^	24	7%
[M + H]^+^	255	74%
Not recognized by algorithm	51	15%
Total	345	Derivatized standards

**Table 4:** Examples of false-negative annotations of molecular species that were missed by the automatic algorithm but rationalized as novel ion species [M + TMS]^+^.

	Observed *m*/*z*	Theoretical	Mass Error [mDa]	Ion Species
3,4-dihydroxy-phenylacetic acid	384.1612	384.1608	−0.4	[M]^+^ 3TMS
	369.1377	369.1374	−0.3	[M − CH_3_]^+^ 3TMS
	413.2004	413.2000	−0.4	[M + C_2_H_5_]^+^ 3TMS
	425.1996	425.2000	0.4	[M + C_3_H_5_]^+^ 3TMS
	457.2088	457.2082	−0.6	[M + TMS]^+^ 3TMS
phosphoric acid	315.1031	315.1033	0.2	[M + H]^+^ 3TMS
	299.0719	299.0720	0.1	[M − CH_3_]^+^ 3TMS
	343.1345	343.1346	0.1	[M + C_2_H_5_]^+^ 3TMS
	355.1342	355.1346	0.4	[M + C_3_H_5_]^+^ 3TMS
	387.1428	387.1428	0.0	[M + TMS]^+^ 3TMS
2,5-dihydroxy-phenylacetic acid	384.1608	384.1608	0.0	[M]^+^ 3TMS
	369.1374	369.1374	0.0	[M − CH_3_]^+^ 3TMS
	413.1995	413.2000	0.5	[M + C_2_H_5_]^+^ 3TMS
	425.1985	425.2000	1.5	[M + C_3_H_5_]^+^ 3TMS
	457.2082	457.2082	0.0	[M + TMS]^+^ 3TMS

**Table 5:** Summary results for 289 TMS compounds with automatically recognized molecular ions.

Correct Formula	Molecular Ion Species	[M − CH_3_]^+^	W/Isotope Pattern
No hit	7.9%	6.9%	12.8%
Top-10	91.7%	93.1%	87.2%
Top-5	87.6%	91.0%	83.4%
Top-3	83.1%	86.9%	78.6%
Top hit	60.7%	71.4%	59.0%

## Data Availability

Chemical ionization mass spectra and codes are freely available at GitHub (https://github.com/Shunyang2018/EICI), (accessed on 11 August 2023).

## Supplementary Materials

The following supporting information can be downloaded at https://www.mdpi.com/article/10.3390/metabo13080962/s1: Supplementary Data S1 with worksheet 1: result data; worksheet 2: summary statistics.
